# Supplementary material for: Patterns of Intron Gain and Loss in Fungi
Source: PLoS Biol. 2004 Nov 30;2(12):e422. doi: 10.1371/journal.pbio.0020422 (PMC532390; doi:10.1371/journal.pbio.0020422)
Supplement: Table S1 — Also available at http://genes.mit.edu/NielsenEtAl/. (4.3 MB ZIP). [file pbio.0020422.st001.zip › NielsenEtAl/html/296.html]

AN8668.1.NCU09094.1.MG02761.1.FG08598.1


```
 CLUSTAL W (1.82) Multiple Sequence Alignments - Introns Inserted


Sequence 1: NCU09094.1	352 aa
Sequence 2: FG08598.1	342 aa
Sequence 3: MG02761.1	345 aa
Sequence 4: AN8668.1	375 aa
Alignment Length: 386 aa
Number Identitical Residues: 98 aa
Alignment Score (without introns) 5720


MG02761.1 	MGSSVRKKKEKKKDFQ0KTKLKVGKAKEKPASFTDTSFKSRA1IVVAQSSLTVEG-PNPV
NCU09094.1	MGSSNKKKKEKKKDFN0KAKLKVGKAKAKAANFTDTSFKSKS1IVVNQHTLAALDGVDLV
FG08598.1 	MGSSNKKKREKQKDFQ0KPKFKVGKDKAKASNFTDTSFKSRA1IVMGHQSLSAVA-PDVV
AN8668.1  	MGASTKRKKEKQKDFQ0KPKLKVGKAKAKPDNFTDTSFKSKA1ITLNQQSLTLTA-PSAN
          	**:* ::*:**:***: *.*:**** * *. .********:: *.: : :*:     .  

MG02761.1 	EQFKHYLSLASTSRSENQRRDALAFLTTQLSHQPPNNPVG--TPAILAKLLPLISDSNSG
NCU09094.1	GLFKQHLNQAINSKSDKLRQEALVQLTKDLSSKPIFNPVG--VPNLLTKLLPLITDSVAN
FG08598.1 	QQFRHNLSLASSSKSDKQRREALAYLTSQLSSEPPVNPVG--THAVLVKLLPLISDSSTP
AN8668.1  	TQFSHHLSLLS-SKSDSQRRDSLAHLTTTLTSQPTHLPPPQPVSVILPSLLPLILDSNAS
          	  * : *.    *:*:. *:::*. **. *: :*   *  ...  :* .***** ** : 

MG02761.1 	VRTQLYKLLQAFPSADIQPQVERISMYVRAGMTHLSADIRDDTLSIMEWLLEVAGSEVVS
NCU09094.1	VRTNFLKLLRALPPSDVAPHVEKILMYIRGGMTHLSTEIRSDTLSVLDWLIDVCPDETVS
FG08598.1 	VRSQLLKLFRELPSDEVRHSVEQAIMFVRAGMTHLSADISNDSLGVMEWLLDVAENDLVV
AN8668.1  	VRANLLKLLRALPQHDVKDHVGTLMPYIRAGMTHLAAEVRVSSVEVLGWLVSIAGDEVVG
          	**::: **:: :*  ::   *     ::*.*****::::  .:: :: **:.:. .: * 

MG02761.1 	CAGGWMKTLNVFAAMLGWSSIV-------AANAAAAAGSAPRNKGWTSAPKSTFG--AAK
NCU09094.1	CPGGWLKTMNSFSSMLGWNPSV-------ASTLSVKG--------WTSATKTSLNKVSKK
FG08598.1 	CPGGWVKTLSSFCAMMGW-----------ALKIPKAG--------WSSGTKSGL---SAK
AN8668.1  	VAGGWVKTLNCFLSVLGWHVAAKSKWTSLATSTSTSASGSGNGTGSSTSGKASYGKAGAK
          	 .***:**:. * :::**    .:. :: * . .  ...:  ... ::. *:  .. . *

MG02761.1 	GGASYARQLLALAKFLEIGFRPEATGSGG-----QRTLWNSLYRLPRT-----------S
NCU09094.1	NGEAQAKQITTLAKFLEAGFRPETPLPYD-----EQRYWDSIYRMPTT-----------P
FG08598.1 	GAATYARQIATLSRFLEAGLRPEAEIPED-----ESEIWDNLYRIPQD-----------S
AN8668.1  	G-RPQVRFLTILADFLDAGIGSSGSQSMDTTADSASESENQYCTFPITTFASHLIPSNFA
          	.  . .: :  *: **: *: ..   . .:::.:     :.   :*  : ::   .:. .

MG02761.1 	NPFGYLNLFGAPRDEDSEIYADREDRQRVFSRK-WHAVITKGLEEAKKEGGAVGRAGAVL
NCU09094.1	NPFAYLNLWGAQRDEDGEMYPDRISRQQVFERK-WRAAIKTGVMGAKQEGGVIGRAASVL
FG08598.1 	NAFEYLNLYGTRRDEEGEMYPSRDARQRVFERR-FLEAVLKGADQAKKEGGATGRAAAGL
AN8668.1  	SPYLTLNLFGTPRDEEGEMYETREDRWRVFVARGFLGAVERGLETARGEGGEVGRVSALA
          	..:  ***:*: ***:.*:*  *  * :**  :.:  .:  *   *: ***  **..:  

MG02761.1 	NQTLKDG-------LADYDDHVL-----
NCU09094.1	DKVLRTAEEGGKKVVEEQKIEEVEEAEA
FG08598.1 	DQVLQNG-MGEYESSTAMDTQDLLSLW-
AN8668.1  	AKVLRNA-------RDDLEGDGFGDEL-
          	 :.*: .           . . . .
```
